# Broad-Scale Latitudinal Variation in Female Reproductive Success Contributes to the Maintenance of a Geographic Range Boundary in Bagworms (Lepidoptera: Psychidae)

**DOI:** 10.1371/journal.pone.0014166

**Published:** 2010-11-30

**Authors:** Marc Rhainds, William F. Fagan

**Affiliations:** 1 Natural Resources Canada, Canadian Forest Service - Atlantic Forestry Centre, Fredericton, New Brunswick, Canada; 2 Department of Biology, University of Maryland, College Park, Maryland, United States of America; California Academy of Sciences, United States of America

## Abstract

**Background:**

Geographic range limits and the factors structuring them are of great interest to biologists, in part because of concerns about how global change may shift range boundaries. However, scientists lack strong mechanistic understanding of the factors that set geographic range limits in empirical systems, especially in animals.

**Methodology/Principal Findings:**

Across dozens of populations spread over six degrees of latitude in the American Midwest, female mating success of the evergreen bagworm Thyridopteryx ephemeraeformis (Lepidoptera: Psychidae) declines from ∼100% to ∼0% near the edge of the species range. When coupled with additional latitudinal declines in fecundity and in egg and pupal survivorship, a spatial gradient of bagworm reproductive success emerges. This gradient is associated with a progressive decline in local abundance and an increased risk of local population extinction, up to a latitudinal threshold where extremely low female fitness meshes spatially with the species' geographic range boundary.

**Conclusions/Significance:**

The reduction in fitness of female bagworms near the geographic range limit, which concords with the abundant centre hypothesis from biogeography, provides a concrete, empirical example of how an Allee effect (increased pre-reproductive mortality of females in sparsely populated areas) may interact with other demographic factors to induce a geographic range limit.

## Introduction

Understanding how species' geographic distributions arise and are maintained constitutes one of the central goals of ecology. The ‘abundant center' hypothesis from biogeography [1] predicts that local population density should decline as one moves from the core of a species' distribution toward the outer fringes, but many ecological mechanisms could give rise to such a pattern. Indeed across species, a broad array of causal factors are known to influence the positions and characteristics of geographic boundaries, but most studies, and especially those dealing with terrestrial animals, have evaluated only one factor at a time in isolation from other determinants [2]. Even among insects, where spatially replicated populations are often more tractable than in other animals, few studies have documented broad scale variation in reproductive success, nor how such variation may limit distribution range [3], [4]. This is unfortunate because spatial variation in birth rate (reproduction) is probably the most critical determinant of geographic range boundaries [2].

Premature mortality of adult females (when females die before they lay their full complement of eggs) has long been known as a determinant of insect population dynamics [5], [6], [7], but the demographic consequences of reproductive failure at low population density (“demographic” Allee effect) [8] have been documented for few insect species [9], [10]. Theoretical models predict that a demographic Allee effect can contribute to maintenance of distributional range both with [11] and without [12] a strong environmental gradient. However, neither scenario has received strong empirical support.

Efforts to understand the structure, maintenance, and dynamics of animal range boundaries in a synthetic way are currently hamstrung by the lack of a model system for which multiple demographic parameters can be concurrently estimated in natural populations starting in the interior of a species range and moving out toward the edge of distribution [2]. Here, we answer the call for a model system for the study of geographic range limits. The bag-centred lifestyle of bagworms (Lepidoptera: Psychidae) makes them ideal animals for investigating geographic variation in demography. Multiple components of female fitness can be assessed using pre- and postmortem dissection of bags, including mortality during the pupal stage, timing of adult stage, mating success of adults, fecundity, overwintering survival of eggs, and reproductive output (Table 1). We provide herein a clear demonstration of how the interplay among a variety of demographic factors, including a striking spatial gradient in reproductive success, contributes to bagworm' geographic range boundary.

**Table 1:** Fitness Estimates.

Fitness component	Classification of Individuals	Equation
Survival during pupal stage (SP)	lp = live pupadp = dead pupaea = emerged adult	SP = (lp + ea/(lp + dp + ea)
Mating success (MS)	m = mated femaleu = unmated female	MS = m/(m+u)
Fecundity (FEC)	w = egg biomass	FEC ≈ w
Overwintering survival of eggs (OWS)	lo = live offspringdo = dead offspring	OWS = lo/(lo + do)
Reproductive output (RO)		RO = MS * FEC

Methodology used to estimate fitness parameters of female *Thyridopteryx ephemeraeformis* (adapted from Rhainds *et al.* 2008).

## Materials and Methods

### Study system

The bagworm *Thyridopteryx ephemeraeformis* (Haworth) is a univoltine, polyphagous moth widespread in the United States. Throughout its range, *T. ephemeraeformis* is broadly distributed as a pest in urban and agricultural landscapes on ornamental trees, predominantly juniper (*Juniperus sp*) and arborvitae (*Thuja occidentalis*).

*Thyridopteryx ephemeraeformis* possesses a suite of life history traits that make it an ideal candidate for a holistic approach to understanding what factors set and maintain range limits. Females are flightless as adults and reproduce within their bags, two traits that greatly facilitate studies of lifetime reproductive success and spatial population dynamics [13], [14]. The bags are conspicuous on their host plant and infestations tend to occur in discrete patches on isolated plants or clusters of plants [14], thus facilitating sampling of local population even at low population density. Populations can be sampled along a broad latitudinal range (32–42°N) in the Midwest, but the species features a distinct geographic limit corresponding to northern Indiana that apparently has been stable for decades [15].

First instars construct a self-enclosing bag from host-plant material and enlarge this bag throughout their development. Upon completion of feeding, larvae tightly attach their bag to the host plant to pupate. Adults emerge in the fall. Males, which are typical winged moths, actively forage for sexually receptive females. The females are paedomorphic (neotenous), flightless, and do not leave their bag before the end of their life. Females attract mates during a ‘calling stage' in which they disseminate setae impregnated with pheromone. Shortly after mating, the female oviposits a single clutch of eggs inside her pupal case and bag [16], [17]; upon oviposition, the females drop to the ground and die. Females that fail to mate do not oviposit and eventually die within their bag, usually outside of the pupal case. The eggs laid by mated females overwinter inside the maternal bag, and neonates emerge in the spring.

### Reproductive success of females for the 2008 reproductive season

**Figure 1:**
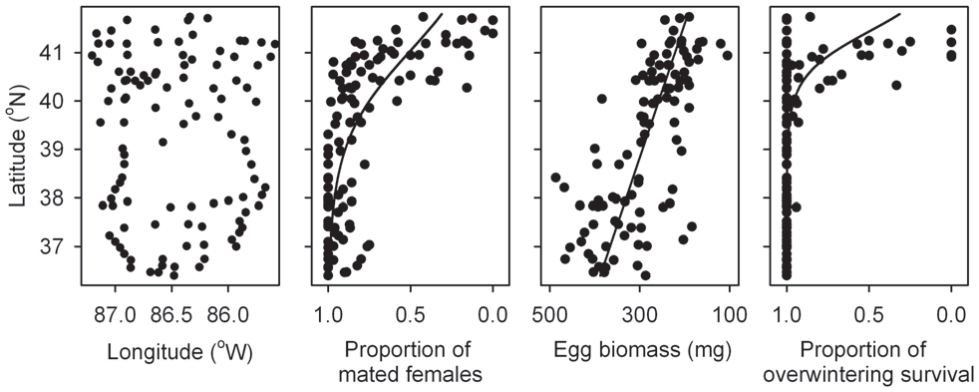
Female fitness in 2008. Spatial distribution of host plants of bagworms, *Thyridopteryx ephemeraeformis*, sampled in Indiana, Kentucky, and Tennessee to determine multiple components of female fitness during the 2008 bagworm generation (Table 1). Latitude (*x*) significantly affected the proportion of mated females [logistic model with 110 sites, *y* = e ^(43.3−1.05 *x*)^/(1+e ^(43.3–1.05 *x*)^), *χ* = 393.46, *P*<0.0001], fecundity [linear model with 100 sites (excluding sites with none or <3 overwintered egg masses), *y* = 1698−36.0 *x*, *r^2^* = 0.496, *P*<0.0001] and the proportion of overwintering survival [logistic model with 104 sites (excluding sites with <3 egg masses sampled), *y* = e ^(89.3−2.16 *x*)^/(1+e ^(89.3−2.16 *x*)^), *χ* = 104.41, *P*<0.0001).

Bagworm bags were sampled in March and April 2009, before the hatching of neonates. Study sites were located by driving through Indiana, Kentucky, and Tennessee along a 155 km wide north-south corridor (Fig. 1) and inspecting junipers for the presence of bagworms. Approximately 50 bags were collected on infested junipers at each of 110 sites. The bags were dissected to determine the mating status of females and the weight of egg masses. Postmortem assessments of the bags were conducted to evaluate the mating status of females based on the presence or absence of eggs (mated and unmated females, respectively) inside the pupal cases of females that had emerged as functional adults [18]. Adult emergence was diagnosed using as criteria the anterior split of the female pupal case and the presence of pheromone-impregnated setae in the lower portion of the bag.

At the time of sampling in early spring, all egg masses laid by mated females appeared healthy (whitish colour, smooth shape). To determine whether the eggs had survived the winter, pupal cases with egg masses were individually marked by location, kept in Solo® cups on a laboratory bench, and monitored daily to determine hatching of early instars. Early instars hatched over a 12 week period from the time of collection to 1 June. After a period of 7 days without emergence, the remaining egg masses that did not yield live larvae were visually inspected. All unhatched eggs had shrunk and turned black, indicative of overwintering mortality [19]. For each site, the mating success of females and proportion of females that produced live progeny (*i.e.*, those females whose eggs overwintered successfully to hatch) were estimated as described in table 1.

### Reproductive success for the 2009 reproductive season

**Figure 2:**
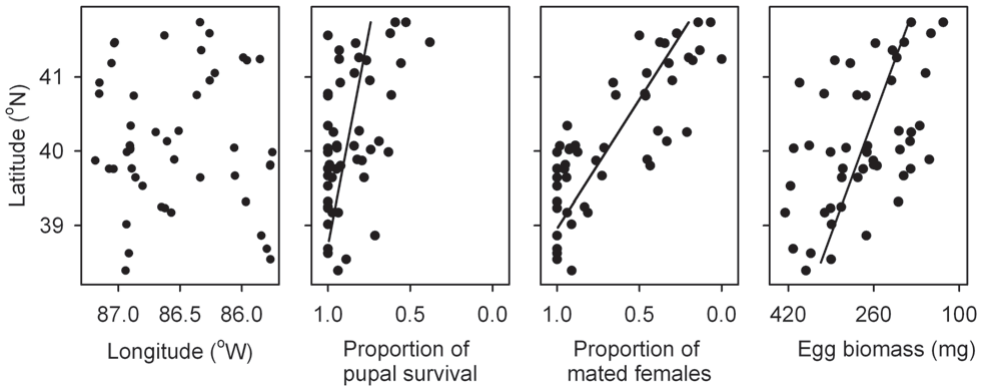
Female fitness in 2009. Spatial distribution of host plants of bagworms, *Thyridopteryx ephemeraeformis*, sampled in Indiana to determine multiple components of female fitness during the 2009 bagworm generation (Table 1). Latitude (x) significantly affected proportions of pupal survival [linear model with 50 sites, *y* = 4.33−0.086 *x*, *r^2^* = 0.257, *P* = 0.0002] and of mated females [linear model with 50 sites, *y* = 12.2−0.29 *x*, *r^2^* = 0.655, *P*<0.0001], as well as fecundity [linear model with 49 (excluding one site with no egg mass sampled), *y* = 2315−50.8 *x*, *r^2^* = 0.305, *P*<0.0001].

Sampling was conducted in Indiana (Fig. 2) throughout the emergence period of adults in late summer and early fall (26 sites for arborvitae and 24 sites for juniper). Sampling was initiated at the onset of pupation and terminated when all females had emerged (10 August to 18 November). Because the sites were distributed across a broad latitudinal range, they could not all be sampled on the same day. Each sampling interval lasted 3–4 days and gaps between sampling intervals lasted 5–10 days. Sampling at a given site ceased when all females had emerged. For each site and sampling interval, between 5 and 34 females (usually >10) were collected during the emergence period, depending on the relative availability of bagworms. Females were removed from their bag and classified as either in the pupal stage (subclassified as live or dead pupae) or emerged adults (subclassified as mated, unmated or calling females). The weight of egg masses laid by mated females was determined for different sites and sampling intervals. Female survival during the pupal stage, mating success, and reproductive success were evaluated at different sites using the equations listed in table 1.

### Spatial distribution of bagworms

**Figure 3:**
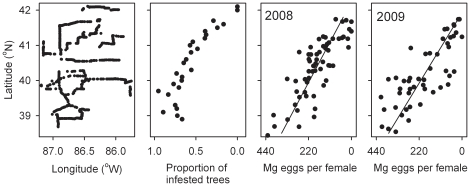
Female fitness in 2008–2009. Spatial distribution of host plants of bagworms, *Thyridopteryx ephemeraeformis*, sampled in Indiana and south Michigan to determine the presence or absence of bagworms (table 3). For comparison, the effect of latitude (x) on female reproductive output (y, in mg eggs; see Table 1) is depicted in 2008 (*y* = 4059−96.1 *x*, *r^2^* = 0.662, *P*<0.0001, *N* = 61 sites) and 2009 (*y* = 4612−110.0 *x*, *r^2^* = 0.652, *P*<0.0001, *N* = 50 sites).

The distribution of bagworms was assessed in 2009 in Indiana and southern Michigan (Fig. 3) by driving along state roads and recording the presence or absence of bagworms on all junipers or arborvitae sighted within 10 to 15 m from each side of the road. In total, the presence or absence of bagworms was assessed on 1196 potential host plants spread along 983 linear km of transects (Fig. 3). For each 0.1° latitudinal band with >10 trees sampled (27 total bands), the proportion of trees infested with bagworms was estimated.

### Rate of extinction of local populations

Because a substantial number of bagworm larvae remain and develop on their natal host [14], it was assumed that host plants that harbored bagworms during the 2008 generation but had no live larvae in 2009 represent local extinction events. The study was conducted at 56 sites previously sampled in Indiana in March 2008 to determine the reproductive success of females. Each site consisted of a group of trees infested with bagworms that was at least 10 m away from other infested trees, with a distance between sites >2 km. Each site was sampled a second time in June 2009 to determine the presence (sustained infestation) or absence (local extinction) of live larvae on the juniper plants. For each site, an index of between-year reproductive output (RS) was tabulated taking into account the probability that females mated and the probability of overwintering survival of eggs. The probability of local extinction across sites was estimated for two classes of female reproductive output [RS = 0 (complete reproductive failure); RS >0 (some females successfully reproduced)] and four latitudinal classes [<39°N; 39–40°N; 40–41°N; >41°N].

### Data analysis

Statistical analysis was conducted with the SAS statistical package (version 9.1, SAS Institute, Cary, NC). Partition of variance analysis was used to evaluate the variance associated with latitude and longitude (one-degree bands) and host plant (juniper or arborvitae). Linear and logistic regression was used to evaluate the effect of latitude on different parameters of fitness. Unless otherwise stated, all the relationships reported are highly significant (*P*<0.0001).

## Results

Partition of variance analysis indicated that the variance component associated with latitude was consistently larger than that associated with longitude or host plant for all parameters, usually by a factor >5 (Table 2). This variance structure justified our use of latitude as a key variable across which we quantified the fitness parameters of female bagworms.

**Table 2:** Source of variation.

Parameter	Variance component		
	Latitude	Longitude	Host plant
*2008 generation*			
Female mating success	1.000	0.029	
Overwintering survival of eggs	1.000	0.122	
Egg biomass	1.000	0.059	
Reproductive output	1.000	0.038	
*2009 generation*			
Pupal survival	1.000	0.109	0.025
Egg biomass	1.000	0.319	0.118
Mating success	1.000	0.100	0.006
Reproductive output	1.000	0.000	0.021
Distribution of infestation	1.000	0.226	0.073

Partition of variance evaluating the variance component associated with latitude, longitude (both classified as one degree bands), and host plant (arborvitae or juniper) for different parameters of fitness. For all parameters, the highest variance component value was standardised as 1.000, to facilitate comparison of variance effects across fitness parameters.

For the 2008 generation of bagworms, logistic regression revealed a significant latitudinal decline in female mating success and egg overwintering survival, with steep declines at latitudes corresponding to central–northern Indiana (above 39°N for mating success and above 41.5°N for overwintering survival). Female fecundity declined linearly with latitude (Fig. 1).

For the 2009 generation, survival during the pupal stage, female mating success and egg biomass all declined linearly with latitude (Fig. 2). Pupal mortality was primarily associated with Hymenoptera and Diptera generalist parasitoids.

The variation in female reproductive output (mating success * egg biomass; Table 1) in 2008 and 2009 was evaluated using only latitudes above 38.4°N so that the data could be compared for different years. Analysis of covariance revealed a highly significant effect of latitude on reproductive success (*F* = 204.98, *df* = 1,107, *P*<0.0001), but no significance of year either alone (*F* = 0.58, *df* = 1,107, *P* = 0.450) or in combination with latitude (*F* = 0.59, *df* = 1,107, *P* = 0.444). Thus, the latitudinal variation in female reproductive success was robust for the 2008 and 2009 bagworm generations. Female fitness declined linearly with latitude in both years and was particularly low above 41°N (Fig. 3).

The proportion of infested trees declined non-linearly with latitude, exhibiting a steep decline above 41° N; no infested trees (out of 109 sampled) were observed above 42° N (Fig. 3; Table 3). The abundance of potential host plants was relatively constant between 39–41°N and increased above 41°N, thus the northern range limit of bagworms cannot be attributed to a lack of potential host plants. Furthermore, the latitudinal decline in abundance is *not* due to interspecific competition because very few defoliators other than bagworms were observed on arborvitae or juniper in the study area.

**Table 3:** Latitudinal distribution of bagworms.

Latitude (°N)	Length of transect (km)	Host plants per km	Infested plants per km	Proportion of infestation
<39.0	75.6	1.15	0.767	0.667
39.0–39.5	83.6	1.03	0.766	0.744
39.5–41.0	197.8	0.99	0.784	0.791
40.0–41.5	156.4	0.99	0.633	0.639
40.5–41.0	186.6	0.94	0.552	0.589
41.0–41.5	183.5	1.49	0.654	0.440
41.5–42.0	54.7	1.92	0.366	0.190
>42.0	44.3	2.46	0.000	0.000

Relative abundance of potential host plants (arborvitae and juniper) and level of infestation by *Thyridopteryx ephemeraeformis* as a function of latitude in Indiana and southern Michigan.

Local extinction of populations between the 2008 and 2009 bagworm generations was observed at 9 of 56 sites (16.1%). The probability of extinction was 100% (*N* = 4) at sites where females experience complete reproductive failure (RS = 0); all these sites occurred above 41°N (Table 4). At sites where some females reproduced (RS >0), no extinction event was observed below 38°N, and the probability of local extinction was roughly constant further north (13.6–15.4%) (Table 4). The high rate of extinction events above 41°N (6 of 17 sites, or 32.6%) was due to the high proportion of sites where females experienced complete reproductive failures (4 of 17 sites, or 23.5%) (Table 4). Logistic regression revealed a significant increase in the rate of extinction (*y*) as a function of latitude (*x*) [*y* = e^(41.87−0.996*x*)^/(1+e^(41.87−0.996*x*)^), *χ* = 3.98, *P* = 0.046].

**Table 4:** Extinction of bagworm populations.

Latitude (°N)	Reproductive output t	Number of sites	Probability of extinction
<39	>1	6	0.000
39–40	>1	13	0.154
40–41	>1	22	0.136
>41	0	4	1.000
	>1	13	0.154
	Total	17	0.326

Probability of extinction of populations of *Thyridopteryx ephemeraeformis* during the winter of 2008–2009 as a function of latitude and reproductive output of females in parental generation.

t Values of 0 are indicative of complete reproductive failure of females, whereas values >1 indicate that at least some females successfully reproduced.

## Discussion

Demographic Allee effects, defined as positive impacts of density on the *total* fitness of individuals (*e.g.*, high rate of mortality and low mating success in sparse populations), have been hypothesized to strongly influence population dynamics and to help constrain geographic range boundaries [8], [11], [12]. Unfortunately, empirical data that could be used to understand how the interplay between spatial gradients and population demography influences the establishment and maintenance of geographic range limits are rare in terrestrial animals [8], [9]. This lack of data stems, in part, from the difficulty of adequately sampling low population density toward the edge of a species' distributional range and also from the lack of a model animal system for which multiple demographic parameters can be concurrently estimated [2]. We report here extremely low mating success of female bagworms in undisturbed, natural populations toward the edge of the distribution range, including the occurrence of total mating failure (0% mated female) at some sites. Of particular interest are the coincident latitudinal decline in bagworm abundance and female mating success above 41°N, and the apparent robustness of the latitudinal trends in 2008 and 2009. Because restricted mobility of females constrains their mating ability [10], species with flightless females may be particularly susceptible to low female mating success at low population density (mate encounter Allee effect) which may in turn influence the species' distributional range, as reported in the gypsy moth, *Lymantria dispar* [20], [21], [22]. Low vagility may further influence the interface between climate change and geographic range limits [3], [23], particularly when species are unable to keep pace with changing landscapes through dispersal [24].

The abundance of potential host plants *per se* does not set the bagworm's range limit, as indicated by the increasing abundance of junipers and arborvitae with latitude (Table 3) and the absence of latitudinal variation in foliar nutrient content of the two main host plants of bagworms, junipers and arborvitae [25]. Because of the limited dispersal ability of bagworms and the naturally fragmented distribution of their host plants in urban and rural landscapes, we suggest that local reproductive success of females helps drive regional persistence for the species in concert with other demographic factors. Indeed, several components of female fitness declined toward the edge of the bagworms' distributional range, including survival during the pupal stage, mating success, fecundity, and overwintering survival of progeny, resulting in an overall reduction in reproductive success of females at northern locations, and in extreme cases, total reproductive failure. The increasing probability of extinction of local populations with declining female reproductive output (Table 4) indicates that patchy bagworm populations toward the range limit are temporally unstable. Such a demographic structure would be consistent with an “invasion pinning” scenario in which an Allee effect limits spatial spread [12].

The ‘abundant-centre' hypothesis proposes that in a species range, a larger percentage of individuals of a population will be present in the centre of the range, where conditions are more favorable. Likewise, the hypothesis proposes a reduced density near the edge of a species range due to the interplay between numerous biotic and abiotic aspects of the habitat that worsens or becomes more intense as a species range boundary is approached [26], [27], [28]. In some cases, researchers question the validity of the abundant-centre hypothesis on the ground that apparent empirical support stems from reduced sampling near the edge of species range [27], [28]. However, in other cases -such as the present study- the sampling designs for assessing population density across space are robust, and evidence for an abundant centre (and low density edges) is strong [29], [30], [31]. Predictive models suggest that the interplay between dispersal and demography can result in species that are 2 to 30 times denser in the centre of the range than at the edges [32]. If a species is going to be subject to Allee type dynamics, the likelihood for or intensity of such effects would be much greater in the vicinity of the range boundaries where densities are lower.

Several mechanisms may be simultaneously at work to restrict the reproductive output of female bagworms toward the edge of the distribution range, including an increased abundance of generalist pupal parasitoids (the most common natural enemies of bagworm pupae), low summer temperatures and short growing seasons (which together restrict the body size and fecundity of females; 33), low population density (which constrains female mating success), and low winter temperatures (which elevate egg mortality). Teasing apart the relative importance of these factors for the maintenance of the bagworms' geographic range limit will require a detailed population model that is parameterized from field data.
